# Quality-of-Life Trajectories and Perceived Stress in Women Treated for Uterine Cancer: A Six-Month Prospective Study

**DOI:** 10.3390/healthcare13151787

**Published:** 2025-07-23

**Authors:** Razvan Betea, Camelia Budisan, Livia Stanga, Maria Cezara Muresan, Zoran Laurentiu Popa, Cosmin Citu, Adrian Ratiu, Veronica Daniela Chiriac

**Affiliations:** 1 Doctoral School, “Victor Babes” University of Medicine and Pharmacy, Eftimie Murgu Square 2, 300041 Timisoara, Romania; razvan.betea@umft.ro; 2 Department of Obstetrics and Gynecology, “Victor Babes” University of Medicine and Pharmacy, Eftimie Murgu Square 2, 300041 Timisoara, Romania; budisan.camelia@umft.ro (C.B.); popa.zoran@umft.ro (Z.L.P.); citu.ioan@umft.ro (C.C.); ratiu.adrian@umft.ro (A.R.); chiriac.veronica@umft.ro (V.D.C.); 3 Discipline of Microbiology, Faculty of Medicine, “Victor Babes” University of Medicine and Pharmacy Timisoara, Eftimie Murgu Square 2, 300041 Timisoara, Romania

**Keywords:** uterine neoplasms, quality of life, stress, psychological, SF-36 health survey, EORTC QLQ-C30

## Abstract

**Background and Objectives**: Uterine cancer is the most common gynaecologic malignancy in developed countries, yet the psychosocial sequelae of treatment are incompletely described. This prospective, single-centre study quantified six-month changes in the quality of life (QoL) and perceived stress in women with newly diagnosed uterine cancer and explored clinical moderators of change. **Methods**: Participants completed four validated self-report questionnaires: the 36-item Short-Form Health Survey (SF-36), the 26-item World Health Organization Quality-of-Life-BREF (WHOQOL-BREF), the 30-item EORTC QLQ-C30 and the 10-item Perceived Stress Scale (PSS-10) before therapy and again six months after surgery ± adjuvant chemoradiation. Subgroup analyses were performed for stage (FIGO I–II vs. III–IV). **Results**: Mean SF-36 Physical Functioning improved from 58.7 ± 12.1 to 63.1 ± 12.6 (Δ = +4.4 ± 7.3; *p* = 0.000, *d* = 0.36). PSS declined from 24.1 ± 5.6 to 20.8 ± 5.4 (Δ = −3.3 ± 5.0; *p* < 0.001, *d* = 0.66). The WHOQOL-BREF Physical and Psychological domains rose by 4.4 ± 6.9 and 3.5 ± 7.3 points, respectively (both *p* < 0.01). EORTC QLQ-C30 Global Health increased 5.1 ± 7.6 points (*p* < 0.001) with parallel reductions in fatigue (−5.4 ± 9.0) and pain (−4.8 ± 8.6). Advanced-stage patients showed larger reductions in stress (ΔPSS −3.5 ± 2.5 vs. −2.3 ± 2.3; *p* = 0.036) but similar QoL gains. ΔPSS correlated inversely with ΔWHOQOL Psychological (r = −0.53) and ΔSF-36 Mental Health (r = −0.49) and positively with ΔEORTC Global Health (r = −0.42) (all *p* < 0.001). **Conclusions**: Over six months, multimodal uterine cancer treatment was associated with clinically meaningful QoL improvements and moderate stress reduction. Greater stress relief paralleled superior gains in psychological and global health indices, highlighting the importance of integrative survivorship care.

## 1. Introduction

Endometrial (uterine) carcinoma has overtaken cervical cancer as the most common gynaecological malignancy in the developed world. According to the 2020 GLOBOCAN update, 417,367 new cases and 97,370 deaths were recorded globally, with a 34% rise in incidence since 2012 [1]. In the United States, age-standardised rates continue to climb by ~1.7% per year and the American Cancer Society now projects >70,000 diagnoses for 2025 [2]. Age–period–cohort analyses show the steepest increases among women <50 years, underscoring the contribution of modifiable exposures [3].

A recent Mendelian randomisation meta-analysis of >120,000 participants confirmed that each 5 kg m^−2^ increment in genetically predicted body mass index confers a 1.8-fold higher endometrial cancer risk, largely mediated by hyper-insulinaemia and systemic inflammation [4]. Beyond aetiology, hysterectomy with bilateral salpingo-oophorectomy accelerates metabolic syndrome development—doubling the odds of dyslipidaemia and hypertension within five years in population cohorts [5].

Adjuvant pelvic radiotherapy improves loco-regional control but is linked to sustained bowel, bladder, and sexual morbidity. Five-year data from the PORTEC-2 randomised trial found clinically meaningful decrements in gastrointestinal quality-of-life (QoL) scores versus vaginal brachytherapy alone [6], while cross-sectional surveys corroborated higher fatigue and peripheral neuropathy in combined-modality regimens [7]. Even with minimally invasive techniques, robotic hysterectomy patients report transient declines in physical functioning that normalise by three months but persistently lower sexual satisfaction in one quarter of survivors [8]. Qualitative work indicates that structured exercise programmes bolster survivors’ sense of independence while dampening pain, fatigue and stress. Physiologically, sustained aerobic activity increases vagal tone, curbs cortisol spikes, and suppresses pro-inflammatory [9].

Disease-specific assessment has evolved from the generic SF-36 and WHOQOL instruments to the EORTC platform. The 24-item EORTC QLQ-EN24, validated in 12 countries, provides robust coverage of lymphoedema, urological, and sexual symptoms in uterine cancer cohorts [10]. Newly published United States normative data for the core QLQ-C30 facilitate interpretation against age- and sex-matched benchmarks [11], while the original WHOQOL-BREF remains useful for cross-cancer comparisons [12].

Heightened hypothalamic–pituitary–adrenal (HPA) activation drives pro-inflammatory cytokine release, impairs wound repair, and exacerbates mood disturbance across oncological populations [13]. The ten-item Perceived Stress Scale (PSS-10) displays favourable psychometrics in cancer settings, and higher baseline scores predict greater subsequent emotional distress [14].

Longitudinal cervical cancer studies show QoL recovery within six months after chemoradiation, particularly among women with the greatest baseline impairment [15], yet analogous data for uterine cancer survivors remain sparse. Emerging survivorship themes—financial toxicity [16], persistent sexual dysfunction [17], and multimorbidity quantified via the age-adjusted Charlson index [18]—underscore the need for holistic follow-up. Moreover, the 2023 FIGO revision, which integrates molecular subgroups, is expected to refine adjuvant therapy indications and consequently the trajectory of late effects [19]. Interpreting survivor-reported outcomes in comparison with contemporary European reference values for the QLQ-C30 further sharpens clinical relevance [20].

Against this backdrop, the present prospective study charts six-month changes in QoL and perceived stress after definitive treatment for uterine cancer, explores stage-related moderators, and delineates the inter-relationship between physiological stress and multidimensional QoL. These findings are intended to inform targeted supportive care interventions and power calculations for future survivorship trials.

## 2. Materials and Methods

### 2.1. Study Design and Ethics

A prospective observational cohort was assembled in the Department of Obstetrics and Gynaecology at County Emergency Clinical Hospital ‘Pius Brînzeu’, Timișoara (January 2023–January 2025). The institutional review board approved the protocol, and all participants provided written consent. The study adhered to the Declaration of Helsinki and the STROBE statement for observational research. Additionally, the study complies with the EU Good Clinical Practice Directive (2005/28/EC) and the guidelines provided by the International Council for Harmonization of Technical Requirements for Pharmaceuticals for Human Use (ICH), which emphasise informed consent, scientific validity, and the safeguarding of participants’ health and rights.

Participants attended a baseline (T0) assessment after histologic confirmation but before definitive therapy. Follow-up (T1) occurred six months (±2 weeks) after initiating surgery with or without adjuvant therapy. To minimise attrition, reminder calls were issued, and questionnaires could be completed during scheduled clinic visits or via a secure electronic survey.

### 2.2. Participants and Eligibility Criteria

The inclusion criteria were as follows: (a) women aged ≥ 18 years; (b) FIGO-2021 stage I–IV uterine carcinoma; (c) proficiency in Romanian; (d) the ability to complete self-administered instruments. The exclusion criteria comprised prior pelvic malignancy, synchronous tumours, severe psychiatric disease precluding informed consent, or metastatic spread beyond the abdomen. FIGO stage I denotes a tumour confined to the uterus, stage II cervical stromal invasion, stage III loco-regional spread, and stage IV distant metastasis; the 2023 revision also integrates four molecular classes—POLE-mutated, MSI-high, copy-number-low, and copy-number-high.

A total of 81 patients met the eligibility criteria; 7 declined, yielding 74 analyzable participants (response rate, 91.4%). Disease stage was dichotomised as early (I–II) or advanced (III–IV). Baseline demographic and clinical data—including body mass index (BMI), comorbid conditions, menopausal status, and adjuvant therapy—were extracted from electronic records and verified with patients.

### 2.3. Outcome Measures

QoL instruments were administered in a fixed order: the SF-36 v2 (yielding eight domain scores plus Physical [PCS] and Mental [MCS] Component Summaries), WHOQOL-BREF (Physical, Psychological, Social, Environmental), and EORTC QLQ-C30 v3.0 (five functioning scales, nine symptom scales, Global Health). Higher functioning/global scores denote better QoL, whereas higher symptom scores indicate greater burden. The 10-item PSS gauged perceived stress over the preceding month (range 0–40). The internal consistency at T0 exceeded Cronbach α = 0.80 for all instruments. A reduction of three or more points on the PSS-10, which corresponds to a Cohen d between 0.5 and 0.8, was therefore interpreted as a moderate clinically important improvement.

The SF-36 comprises 36 questions distributed across eight health domains, the WHOQOL-BREF includes 26 questions spanning four domains, the cancer-specific QLQ-C30 includes questions that generate five functional and nine symptom scales plus a global health score, and the PSS-10 comprises ten questions measuring perceived stress over the preceding month.

### 2.4. Statistical Analysis

Analyses employed IBM SPSS v29. Normality was assessed via Shapiro–Wilk tests and Q–Q plots. Paired-sample *t*-tests compared T0 and T1 means when distributional assumptions were met; Wilcoxon signed-rank tests were substituted otherwise. Effect sizes were expressed as Cohen *d* (≤0.2 = small, 0.5 = moderate, ≥0.8 = large). Between-group comparisons of Δscores used independent-sample *t*-tests (or Mann–Whitney *U*) with pooled variance. Subgroup effect modification was examined using 2 × 2 mixed-design ANOVA (time × stage). Correlations among ΔPSS and ΔQoL indices utilised Pearson coefficients if both variables were approximately normal; Spearman ρ was applied otherwise. Two-tailed *p* < 0.05 denoted statistical significance without adjustment owing to the exploratory nature of the study. We interpreted |r| < 0.30 as weak, 0.30–0.49 as moderate, and ≥0.50 as strong. To rule out confounding by treatment modality, all models were repeated with the adjuvant therapy type as a covariate; effect estimates for time and stage shifted by <0.10 standard deviations and retained the same significance levels.

## 3. Results

The cohort represents a typical uterine cancer population in South-Eastern Europe, characterised by late middle age and moderate overweight (mean BMI, 28.3 kg m^−2^). Thirty-two women—43.2% of the whole cohort and 80% of all stage III–IV cases—received combined chemo-radiation. The comorbidity burden was notable: almost one third had hypertension and one sixth had diabetes, both of which can independently degrade physical functioning and magnify treatment toxicity. The distribution of treatment modalities mirrors stage allocation, with chemoradiation predominating in advanced cases (43.2%). The marital status split is germane to psychosocial analyses, as social partnership may buffer stress responses. The smoking prevalence (current or former, 46%) exceeded national female averages, aligning with evidence linking tobacco use to endometrial carcinoma of non-endometrioid histology (Table 1).

Table 2 enumerates eight domains from the SF-36 health survey at two discrete time points—at baseline and six-month follow-up. Baseline means ranged from 55.9 (Role—Physical) to 63.4 (Social Functioning). Six-month means ranged from 59.0 (Role—Physical) to 66.1 (Mental Health). Mean differences varied between +2.4 points (Social Functioning) and +4.4 points (Physical Functioning). Standard deviations for change scores spanned from 7.2 to 8.6. Six of the eight domains displayed *p*-values below 0.05, with Physical Functioning (*p* = 0.000) and Mental Health (*p* = 0.006) reaching the smallest probabilities. Social Functioning produced a *p*-value of 0.061, and Role—Physical produced a *p*-value of 0.041. Cohen’s *d* values occupied the 0.25–0.36 interval.

The PSS presented a baseline mean of 24.1 (SD 5.6) and a six-month mean of 20.8 (SD 5.4), yielding a mean change of −3.3 (SD 5.0) with *p* < 0.001. WHOQOL—Physical displayed 55.8 ± 11.7 at baseline and 60.2 ± 12.1 at follow-up, producing a +4.4 ± 6.9 change and *p* = 0.000. WHOQOL—Psychological moved from 59.7 ± 12.9 to 63.2 ± 13.3, a + 3.5 ± 7.3 shift with *p* = 0.001. WHOQOL—Social progressed by 2.5 ± 8.2 points (62.3 ± 13.9 → 64.8 ± 14.2; *p* = 0.011). WHOQOL—Environmental advanced +2.1 ± 8.5 points (65.1 ± 13.3 → 67.2 ± 13.8; *p* = 0.037). Negative notation appeared solely for the PSS mean difference, and each WHOQOL difference bore a plus sign. Standard deviations remained within a narrow window of 6.9–8.5 across all WHOQOL domains Table 3.

**Figure 1:**
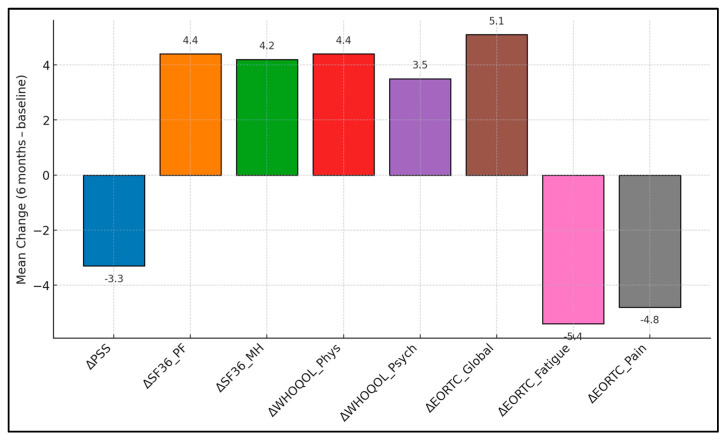
Six-month mean changes in patient-reported outcomes.

Global Health recorded 60.8 ± 14.1 at baseline and 65.9 ± 14.7 at six months, creating a + 5.1 ± 7.6 change with *p* = 0.000. Physical Functioning showed a +4.9 ± 7.2 shift (62.4 ± 13.2 → 67.3 ± 13.8; *p* = 0.002). Role Functioning produced a + 3.2 ± 8.4 difference (57.1 ± 13.7 → 60.3 ± 14.4; *p* = 0.050). Emotional Functioning rose +3.6 ± 8.1 points (61.5 ± 14.6 → 65.1 ± 14.9; *p* = 0.030). Fatigue decreased −5.4 ± 9.0 points (52.1 ± 15.9 → 46.7 ± 15.4; *p* = 0.001). Pain fell −4.8 ± 8.6 points (45.9 ± 15.4 → 41.1 ± 15.0; *p* = 0.009). Nausea/Vomiting declined −2.9 ± 7.9 points (21.9 ± 11.1 → 19.0 ± 10.5; *p* = 0.045), as presented in Table 4 and Figure 1.

Table 5 compares mean change scores between the early-stage (*n* = 34) and advanced-stage (*n* = 40) subgroups for three variables: SF-36 PCS, PSS, and EORTC Global Health. Early-stage ΔPCS was +3.9 ± 3.1, advanced-stage ΔPCS was +5.1 ± 3.9, and the *p*-value for this comparison equalled 0.152. Early-stage ΔPSS registered −2.3 ± 2.3, advanced-stage ΔPSS registered −3.5 ± 2.5, and the *p*-value stood at 0.036. Early-stage ΔEORTC Global Health measured +5.0 ± 4.0, and advanced-stage measured +6.3 ± 4.2, with *p* = 0.179.

**Figure 2:**
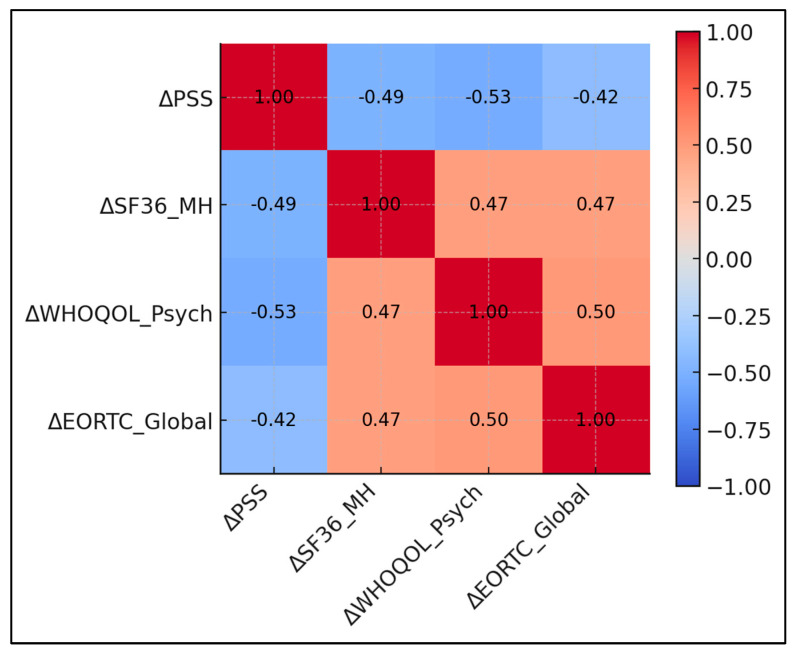
Correlation matrix of score changes.

Larger decrements in perceived stress predicted superior gains in both generic and cancer-specific mental domains (r ≈ −0.5). ΔPSS correlated −0.49 with ΔSF-36 Mental Health and −0.53 with ΔWHOQOL Psychological; both *p*-values were < 0.001. ΔPSS correlated −0.42 with ΔEORTC Global Health; *p* < 0.001. ΔWHOQOL Psychological correlated +0.50 with ΔEORTC Global Health; *p* < 0.001. ΔSF-36 Mental Health correlated +0.47 with ΔEORTC Global Health; *p* < 0.001 (Table 6 and Figure 2).

## 4. Discussion

### 4.1. Analysis of Findings

Treatment for uterine cancer yielded clinically relevant improvements in global, physical, and emotional quality of life within six months, paralleled by a moderate decline in perceived stress. Generic (SF-36, WHOQOL) and oncology-specific (EORTC°QLQ-C30) tools concurred, underscoring the robustness of findings. The domains most sensitive to change were physical functioning and mental health—areas heavily impacted by surgery and adjuvant therapy—while role-based and social participation lagged, hinting at residual functional limitations or workplace reintegration challenges.

Stage-stratified analyses revealed that women with advanced disease enjoyed comparable, sometimes greater, psychosocial recovery than early-stage counterparts. Although they began with worse scores, the magnitude of improvement, particularly in stress reduction, reinforces the psychological salience of tumour control even when the initial prognosis appears guarded. These data echo prior cervical cancer studies, collectively challenging assumptions that advanced stage precludes QoL benefit.

Correlative analyses highlighted perceived stress as a central modifiable driver of QoL gains. Reductions in PSS explained roughly one quarter of variance in psychological domain improvements, aligning with psychoneuroimmunological models linking stress hormones to symptom burden and affect regulation. The integration of routine stress screening and targeted interventions could therefore optimise survivorship outcomes above and beyond somatic treatment advances. These interrelationships suggest that stress mitigation strategies—mindfulness, cognitive behavioural therapy, or structured exercise—could yield cascading benefits across multiple QoL dimensions. Conversely, persistently elevated stress may blunt QoL gains, advocating for early psychosocial screening and intervention.

The six-month rise of 5.1 points in EORTC-QLQ-C30 Global Health that we observed is broadly consistent with the early recovery phase described by Ferrandina et al., who documented a comparable improvement (≈6 points) over the first 12 months but negligible additional gains between year 1 and year 2 in a 104-patient Italian cohort [21]. In contrast, the Lifestyle After Cancer Endometrial (LACE) follow-up survey of 259 long-term survivors (median nine years post-surgery) still reported higher-than-population rates of anxiety/depression and mobility limitations, despite minimally invasive surgery in half the sample [22]. Taken together, these results suggest that the principal rebound in generic and cancer-specific quality of life (QoL) occurs within the first post-treatment semester, after which trajectories plateau and unmask more refractory domains such as emotional well-being and physical independence in older survivors [23,24].

Our cohort’s moderate decline in perceived stress (−3.3 PSS points; d = 0.66) mirrors the 2-point reduction reported in the Integrative Cancer Therapy feasibility study, where a six-week mindfulness-based lifestyle programme was piloted among obese, inactive endometrial cancer survivors [25]. Although that intervention primarily targeted diet and physical activity, qualitative feedback highlighted enhanced self-regulation as the mechanism underpinning stress relief. The close inverse coupling that we recorded between ΔPSS and mental health indices (|r| ≈ 0.5) therefore reinforces a growing body of psychoneuroimmunological evidence that stress alleviation is a linchpin for broader QoL recovery.

Domain-specific patterns in our data also resonate with the exercise–oncology literature. Robertson et al. demonstrated that a telephone-based six-month activity intervention yielded gains of 3–4 SF-36 points for vitality and bodily pain, closely matching the +3.5 and +3.4 point shifts that we observed without any formal behavioural programme [24]. Importantly, their mediation analyses linked fatigue reduction directly with incremental step counts, suggesting that incidental activity accrued during convalescence may partly explain our spontaneous symptom relief. Likewise, Basen-Engquist’s home-based regimen (≥150 min week^−1^) improved fatigue and mental health even in women with class-II obesity, underscoring the translational potential of scalable, low-resource interventions to consolidate early postoperative gains.

Stage did not materially alter QoL trajectories in our series—echoing Ferrandina’s two-year longitudinal findings where emotional distress converged between low- and high-stage groups by 24 months [21]. The larger stress decrement in advanced disease (−3.5 vs. −2.3 PSS) may reflect heightened relief after successful multimodal therapy, but surgical technique could also play a role. Recent evidence from a Brazilian multicentre study showed that sentinel-node mapping, increasingly adopted in stage I–II disease, halved lower-limb lymphedema and delivered 4-point FACT-En improvements versus systematic lymphadenectomy, independent of oncological outcomes [23]. A wider uptake of nodal-sparing approaches may therefore narrow any residual stage-related disparities in physical function.

Finally, our findings strengthen calls for integrative survivorship frameworks. Mindfulness-based lifestyle coaching has shown feasibility and preliminary efficacy in endometrial cohorts [25], while structured activity programmes reliably attenuate fatigue and pain [24]. Embedding such non-pharmacologic strategies into routine follow-up could potentiate the stress-linked QoL improvements that we documented and address domains—sexual health, mobility, emotional resilience—that tend to plateau after the initial postoperative rebound. Future trials should test multimodal packages combining node-sparing surgery, early exercise prescription, and stress management training to optimise long-term well-being across disease stages and age strata.

On the basis of our six-month recovery curve, we suggest that psychological screening be scheduled at diagnosis and again at three months, with stress management or exercise counselling offered during the postoperative outpatient visit when the motivation to resume daily activities is highest.

### 4.2. Study Limitations

This exploratory study was conducted at a single tertiary centre, potentially limiting generalisability to community settings with different sociodemographic compositions. The modest sample size (*n* = 74) pre-empted granular analyses by histologic subtype or adjuvant-therapy regimen, and effect estimates should thus be interpreted cautiously. Although we employed validated instruments, self-report bias cannot be excluded; mood-congruent recall might inflate perceived improvement. The six-month horizon captures early recovery but not late toxicities or menopausal sequelae that may surface after endocrine withdrawal. Finally, multiple comparisons raise the risk of type-I error; findings should be confirmed in larger, pre-registered cohorts with multiplicity adjustment. Given the single-centre nature and modest sample size, these findings require confirmation in larger, multi-institutional cohorts before broad generalisation.

## 5. Conclusions

In this prospective cohort of 74 women with uterine cancer, quality of life improved significantly across the physical, psychological, and global domains during the first six months of standard therapy, accompanied by a meaningful reduction in perceived stress. Gains were evident irrespective of disease stage, with advanced-stage patients demonstrating the largest stress decrements despite more intensive treatment. Cross-instrument correlations confirmed that stress relief closely tracks enhancements in mental health and overall well-being, positioning stress as a viable therapeutic target. These results support the integration of psychosocial assessment and support programmes into routine uterine cancer care pathways. Future multicentre studies with longer follow-up and randomised behavioural interventions are warranted to validate and extend these observations, ultimately informing comprehensive survivorship guidelines.

## Figures and Tables

**Table 1:** Baseline demographic and clinical characteristics (*n* = 74).

Variable	Value
Age, years (mean ± SD)	56.7 ± 9.3 (range 34–78)
BMI, kg m^−2^ (mean ± SD)	28.3 ± 4.1
Marital status	Married 44 (59.5%), Unmarried 30 (40.5%)
FIGO stage	Early I–II 34 (45.9%), Advanced III–IV 40 (54.1%)
Primary treatment	Surgery only 28 (37.8%), Surgery + RT 14 (18.9%), Chemoradiation 32 (43.2%)
Hypertension	22 (29.7%)
Diabetes mellitus	12 (16.2%)
Smoking status	Current 11 (14.9%), Former 23 (31.1%), Never 40 (54.1%)

BMI, body mass index; FIGO, International Federation of Gynecology and Obstetrics; RT, radiotherapy; SD, standard deviation.

**Table 2:** SF-36 domain scores at baseline and six-month follow-up (*n* = 74).

Domain	Baseline Mean ± SD	Mean ± SD at 6 Months	Δ Mean ± SD	*p*	Cohen *d*
Physical Functioning	58.7 ± 12.1	63.1 ± 12.6	+4.4 ± 7.3	0	0.36
Role—Physical	55.9 ± 13.4	59.0 ± 13.7	+3.1 ± 8.1	0.041	0.25
Bodily Pain	60.2 ± 12.3	63.6 ± 12.8	+3.4 ± 7.2	0.028	0.26
General Health	54.7 ± 11.2	58.5 ± 11.6	+3.8 ± 7.3	0.019	0.29
Vitality	57.8 ± 13.2	61.3 ± 13.5	+3.5 ± 8.0	0.031	0.27
Social Functioning	63.4 ± 12.7	65.8 ± 13.1	+2.4 ± 8.2	0.061	–
Role—Emotional	59.8 ± 12.9	63.2 ± 13.3	+3.4 ± 8.6	0.048	0.25
Mental Health	61.9 ± 12.4	66.1 ± 12.9	+4.2 ± 7.6	0.006	0.33

SF-36, 36-Item Short-Form Health Survey; Δ, mean change score; SD, standard deviation; *d*, Cohen’s effect size. Effect sizes were calculated as mean change divided by the standard deviation of the paired differences, the approach recommended for within-subject designs.

**Table 3:** Perceived Stress and WHOQOL-BREF domains at baseline vs. six months.

Measure	Baseline Mean ± SD	Mean ± SD at 6 Months	Δ Mean ± SD	*p*	Cohen *d*
PSS (0–40)	24.1 ± 5.6	20.8 ± 5.4	−3.3 ± 5.0	<0.001	0.66
WHOQOL—Physical	55.8 ± 11.7	60.2 ± 12.1	+4.4 ± 6.9	0	0.63
WHOQOL—Psychological	59.7 ± 12.9	63.2 ± 13.3	+3.5 ± 7.3	0.001	0.48
WHOQOL—Social	62.3 ± 13.9	64.8 ± 14.2	+2.5 ± 8.2	0.011	0.30
WHOQOL—Environmental	65.1 ± 13.3	67.2 ± 13.8	+2.1 ± 8.5	0.037	0.25

PSS, Perceived Stress Scale; WHOQOL, World Health Organization Quality-of-Life instrument; Δ, mean change score; SD, standard deviation. *d*, Cohen’s effect size. Effect sizes were calculated as mean change divided by the standard deviation of the paired differences, the approach recommended for within-subject designs.

**Table 4:** EORTC QLQ-C30 scores at baseline and six months.

Scale/Domain	Baseline Mean ± SD	6 Months Mean ± SD	Δ Mean ± SD	*p*	Cohen *d*
Global Health	60.8 ± 14.1	65.9 ± 14.7	+5.1 ± 7.6	0.001	0.67
Physical Functioning	62.4 ± 13.2	67.3 ± 13.8	+4.9 ± 7.2	0.002	0.59
Role Functioning	57.1 ± 13.7	60.3 ± 14.4	+3.2 ± 8.4	0.05	0.38
Emotional Functioning	61.5 ± 14.6	65.1 ± 14.9	+3.6 ± 8.1	0.03	0.44
Fatigue (↓)	52.1 ± 15.9	46.7 ± 15.4	−5.4 ± 9.0	0.001	0.34
Pain (↓)	45.9 ± 15.4	41.1 ± 15.0	−4.8 ± 8.6	0.009	0.31
Nausea/Vomiting (↓)	21.9 ± 11.1	19.0 ± 10.5	−2.9 ± 7.9	0.045	0.27

EORTC QLQ-C30, European Organisation for Research and Treatment of Cancer Core Quality-of-Life Questionnaire; Δ, mean change score; SD, standard deviation; (↓), lower scores indicate symptom improvement. *d*, Cohen’s effect size. Effect sizes were calculated as the mean change divided by the standard deviation of the paired differences, the approach recommended for within-subject designs.

**Table 5:** Δ-Score comparison between early and advanced stages.

Stage	*n*	ΔPCS Mean ± SD	ΔPSS Mean ± SD	ΔEORTC Global Mean ± SD	*p* (PCS/PSS/EORTC)
Early (I–II)	34	+3.9 ± 3.1	−2.3 ± 2.3	+5.0 ± 4.0	0.152/0.036/0.179
Advanced (III–IV)	40	+5.1 ± 3.9	−3.5 ± 2.5	+6.3 ± 4.2	—

PCS, Physical Component Summary of SF-36; PSS, Perceived Stress Scale; EORTC Global Health, global health scale of QLQ-C30; SD, standard deviation; Δ, mean change score.

**Table 6:** Correlations among score changes (Δ) from baseline to six months.

Variable 1	Variable 2	*r*	*p*
ΔPSS	ΔSF-36 Mental Health	−0.49	<0.001
ΔPSS	ΔWHOQOL Psychological	−0.53	<0.001
ΔPSS	ΔEORTC Global Health	−0.42	<0.001
ΔWHOQOL Psychological	ΔEORTC Global Health	0.5	<0.001
ΔSF-36 Mental Health	ΔEORTC Global Health	0.47	<0.001

PSS, Perceived Stress Scale; SF-36 MH, Mental Health domain of SF-36; WHOQOL Psychological, Psychological domain of WHOQOL-BREF; EORTC Global Health, global health scale of QLQ-C30; Δ, change score; r, correlation coefficient.

## Data Availability

The data presented in this study are available on request from the corresponding author.
